# Deciphering the Role of *Shank3* in Dendritic Morphology and Synaptic Function Across Postnatal Developmental Stages in the *Shank3B* KO Mouse

**DOI:** 10.1007/s12264-024-01330-y

**Published:** 2024-12-18

**Authors:** Jing Yang, Guaiguai Ma, Xiaohui Du, Jinyi Xie, Mengmeng Wang, Wenting Wang, Baolin Guo, Shengxi Wu

**Affiliations:** 1 Department of Basic Medicine, School of Medicine, Hangzhou City University, Hangzhou, 310015 China; 2 Department of Neurobiology, School of Basic Medicine, Fourth Military Medical University, Xi’an, 710032 China; 3 Innovation Research Institute, Xijing Hospital, Fourth Military Medical University, Xi’an, 710032 China

**Keywords:** Autism, *Shank3*, Neuronal development, Dendritic development, Synaptic function

## Abstract

Autism Spectrum Disorder (ASD) is marked by early-onset neurodevelopmental anomalies, yet the temporal dynamics of genetic contributions to these processes remain insufficiently understood. This study aimed to elucidate the role of the *Shank3* gene, known to be associated with monogenic causes of autism, in early developmental processes to inform the timing and mechanisms for potential interventions for ASD. Utilizing the *Shank3B* knockout (KO) mouse model, we examined *Shank3* expression and its impact on neuronal maturation through Golgi staining for dendritic morphology and electrophysiological recordings to measure synaptic function in the anterior cingulate cortex (ACC) across different postnatal stages. Our longitudinal analysis revealed that, while *Shank3B* KO mice displayed normal neuronal morphology at one week postnatal, significant impairments in dendritic growth and synaptic activity emerged by two to three weeks. These findings highlight the critical developmental window during which *Shank3* is essential for neuronal and synaptic maturation in the ACC.

**Supplementary Information:**

The online version contains supplementary material available at 10.1007/s12264-024-01330-y.

## Introduction

Autism spectrum disorder (ASD) is a severe neurodevelopmental condition characterized by impaired social interaction and language and communication skills, as well as repetitive behaviors with restricted interests [1, 2]. In recent years, the rising prevalence and significant impact of ASD have garnered considerable attention, prompting efforts to uncover its pathological mechanisms. Although it is well-established that ASD is rooted in neurodevelopmental processes, the specific neural mechanisms that give rise to the condition are not yet fully understood. Consequently, investigating the neural substrates associated with autistic behaviors, particularly from a developmental perspective, has become a pivotal approach to understanding the pathogenesis and potential treatments for ASD.

Both genetic and environmental factors during early development are believed to significantly contribute to the etiology of ASD [3]. Among the numerous genes identified as risk factors [4, 5], *Shank3* gene mutations are recognized as monogenic causes of ASD [6–8]. Animal studies have consistently shown that SHANK3 is crucial for the formation of excitatory synapses and the maintenance of glutamate receptor function, with *Shank3*-deficient mice displaying ASD-like phenotypes [9–12]. Our previous study further showed that *Shank3* deletion in the anterior cingulate cortex (ACC) leads to social behavior deficits, whereas re-expression of *Shank3* in the ACC of *Shank3B* knockout (KO) mice can rescue these ASD-like social behavioral abnormalities [13]. These findings underscore the important role of *Shank3* in the molecular pathogenesis of ASD.

SHANK3 was initially identified as a postsynaptic scaffold protein that organizes synaptic signaling [14, 15]. Subsequent studies have shown that mutations impairing SHANK3 expression or function cause neuronal deficits by primarily impairing synaptic activity [9, 10, 16, 17]. Intriguingly, despite *Shank3* being expressed from a young age in both macaque and human neocortex [18], the bulk of research has focused on its role in adult organisms, leaving a gap in our understanding of its developmental influence on ASD phenotypes. *In vitro*, experiments with cultured rodent neurons have revealed that overexpression of *Shank3* can increase dendritic spine formation [19], while its knockdown reduces spine numbers and alters spine width and length [20]. Using homologous recombination to conditionally inactivate *SHANK3* in human embryonic stem cell lines, a pioneering study showed that *SHANK3* haploinsufficiency impairs dendritic development in human neurons [21]. Furthermore, a previous study found that *SHANK3* deletion in human telencephalic organoids reduces excitatory synapse numbers [22]. These studies collectively provide evidence for *SHANK3's* involvement in neuronal development. However, *in vivo* investigations, particularly those tracking developmental changes, into the early developmental roles of *Shank3* remain scarce. Furthermore, the presence of multiple isoforms of *Shank3* in the brain complicates the study of its functions [16]. Therefore, we sought to explore the impact of *Shank3* on neuronal development using the *Shank3B* KO mouse model. In this model, the disruption of exons 13 to 16, which encode the PDZ domain, results in the complete lack of expression of the alpha and beta isoforms of *Shank3*, as well as a diminished expression of the gamma isoform [12]. This reduction is associated with autistic-like behaviors, enabling us to trace the developmental roots that may underlie the phenotypic manifestations of ASD.

Our investigation into the spatiotemporal expression of *Shank3* in postnatal mice revealed that SHANK3 is highly expressed in the pyramidal neurons of the ACC, motor cortex (M), and somatosensory cortex (S) at various developmental stages. To further investigate the role of *Shank3* in early development, we analyzed the dendritic morphology and changes in synaptic function in *Shank3B* KO mice at different postnatal stages. Using Golgi staining and electrophysiological recording techniques, we found that *Shank3B* KO mice showed normal neuronal morphology at postnatal week 1. However, by 2–3 weeks postnatal, these mice displayed significant deficits in dendritic length, branching, and spine density, along with synaptic functional impairments. These findings suggest that *Shank3* has a dynamic developmental time window during which it is critical for proper neuronal and synaptic maturation in the ACC. This aligns with the concept that early interventions might be crucial for ameliorating ASD symptoms.

## Materials and Methods

### Animals

The care and experimental procedures involving animals in this study were authorized by the Institutional Animal Care and Use Committee of the Fourth Military Medical University and adhered to the NIH Guide for the Care and Use of Laboratory Animals. Mice were kept under controlled conditions at a stable temperature and under a 12-h light/dark cycle, with *ad libitum* access to food and water. Randomization was applied to assign animals to experimental groups. Previously described [23] GAD67-GFP knock-in mice, which were used to indicate the GAD67-positive cells, were bred with *Shank3B* KO mouse strains [12] on a C57BL/6J background. Both male and female mice aged from 1 to 8 weeks were included in experiments, which were conducted by investigators blinded to genotype and group assignment.

### Immunofluorescence

Immunofluorescence was applied as previously described [24]. In brief, mice were anesthetized with isoflurane, perfused with 0.01 mol/L PBS, and their brains were fixed in 4% paraformaldehyde. After post-fixation and sucrose dehydration, 30-μm coronal sections were cut at −20℃ on a microtome (Leica CM1800, Heidelberg, Germany). For mapping, the sections from the rostral to the caudal part of the brain were used. Following PBS washes and blocking with PBS containing 10% normal donkey serum, sections were incubated with mouse anti-SHANK3 (1:200, 75344, Neuromab, UC Davis, USA) overnight at 4℃; for double staining, the following primary antibodies were used: mouse anti-SHANK3 (1:200, 75344, Neuromab), rabbit anti-SHANK3 (1:200, ab264347, Abcam, Cambridge, UK), mouse anti-NeuN (1:500, ZMS377, Millipore, Mass, USA), rabbit anti-GFAP (1:1000, SAB5700611, Sigma, Mass, USA), goat anti-Iba1 (1:200, ab289874, Abcam, Cambridge, UK). Secondary antibodies conjugated with Alexa Fluor (1:1000, A21202, A21206, A31570-31573, Invitrogen/Life Technologies, CA, USA) were applied post-wash. Prepared slides were imaged using a confocal laser scanning microscope with a 10×/0.45 or 20×/0.75 objective (FV1200, Olympus, Tokyo, Japan), focusing on cortical regions from 0 mm to 0.98 mm from bregma, with 3–5 sections analyzed per mouse.

### Western Blot Analysis

Western blot analysis began with perfusion of anesthetized mice, followed by cortical tissue lysis in RIPA buffer containing 10 mmol/L Tris, 150 mmol/L NaCl, 1% Triton X-100, 0.5% NP-40, and 1 mmol/L EDTA (pH 7.4) with a protease inhibitor cocktail (Roche, Basel, Switzerland). Protein concentrations were determined by BCA assay (Thermo Fisher Scientific, Mass, USA), and 30 mg of protein per lane were separated by SDS-PAGE and transferred to PVDF membranes (Millipore). After blocking and overnight incubation with mouse anti-SHANK3 (1:200, 75344, Neuromab), membranes were washed, exposed to secondary antibodies (Jackson), and visualized using an enhanced chemiluminescence detection system (Yeasen Biotechnology, Shanghai, China). Bands were analyzed with ImageJ software.

### Golgi Staining and Morphological Analysis

Mice were anesthetized with isoflurane and perfused with 0.01 mol/L PBS before the entire brain was extracted and immersed in a Golgi-Cox solution for three days at room temperature. This solution contained a mixture of 5% K_2_Cr_2_O_7_, 5% HgCl_2_, and 5% K_2_CrO_4_ in distilled water. The brains were then cut into 200 μm coronal slices on a vibratome, followed by meticulous rinsing in distilled water and a dehydration step in 50% alcohol. The slices were further incubated in an ammonia solution before being rinsed again and treated with 5% sodium thiosulfate in the dark. After these preparatory steps, cortical sections were imaged under an FV1200 confocal microscope with a 559-nm excitation wavelength, and the optical path was set to 'mirror' mode. Pyramidal neurons from the ACC appearing relatively isolated, were selected for detailed morphological analysis. These neurons are distinguished by their pyramidal configuration, featuring a substantial soma. Protruding from the soma is a prominent apical dendrite that extends toward the cortical surface and multiple shorter basal dendrites that typically spread out horizontally. Whole neurons were photographed using a 20× objective lens, while dendritic spines were observed using a 60× objective lens. Three-dimensional images (2048 × 2048 pixels) were captured from both apical and basal dendrites, with 50-μm sections imaged at 0.5-μm intervals along the Z-axis. Following image acquisition, ImageJ software with the Neuron J plugin was applied to delineate neuronal morphology. In addition, the Sholl Analysis plugin was used to quantify the dendritic length, number, and branching of both apical and basal dendrites. For dendritic spine analysis, Imaris software (v.7.7.1, Bitplane, Zurich, Switzerland) was used; confocal images were directly imported into Imaris, and the Filament tracer module in the surpass interface was applied to reconstruct the imaged dendrites and spines. Subsequently, the built-in spine classification module was used to categorize the reconstructed dendritic spines. All exported data were analyzed with GraphPad Prism.

### Quantitative Real-time PCR (RT-PCR)

Total RNA from the murine cortex at different development stages was extracted with TRIzol (400001, Invitrogen) according to the manufacturer’s instructions. cDNA was generated using the RevertAid First Strand cDNA Synthesis Kit (00597742, Thermo Fisher Scientific). The Hieff qPCR SYBR Green Master Mix (No Rox) (11201ES08, Yeasen Biotechnology) was used for mRNA detection on a Roche Light Cycler (384-well format). ΔCt was used to calculate mRNA changes of the target gene and then normalized to the mean Ct value of the housekeeping gene GAPDH. Each reaction was performed three times and the *Shank3* and *GAPDH* primers used were: *Shank3* (Forward 5’-TCCTGAAGGTTCTCCGCAAC-3’, Reverse 5’-CCTGGTTGTAGAGGGCACAG-3’); *GAPDH* (Forward 5’-GGTTGTCTCCTGCGACTTCA-3’, Reverse 5’-TAGGGCCTCTCTTGCTCAGT-3’).

### Slice Electrophysiology

#### Electrophysiology

Whole-cell patch-clamp recording from isolated brain slices was performed as previously described [13, 25]. In brief, mice were deeply anesthetized with isoflurane, and then the animals were transcardially perfused with carbonated (95% O_2_, 5% CO_2_) cutting solution (in mmol/L): 115 choline chloride, 26 NaHCO_3_, 2.5 KCl, 1.25 NaH_2_PO_4_, 0.5 CaCl_2_, 8 MgCl_2_, 10 D-(+)-glucose, 0.1 L-ascorbic acid and 0.4 sodium pyruvate (300 mOsml^−1^). The 300-μm slices containing the ACC were cut on a vibratome (7000 smz-2, Campden Instruments, Leicestershire, UK). Whole-cell patch-clamp recordings were then performed in artificial cerebral spinal fluid (ACSF) (in mmol/L): 119 NaCl, 26 NaHCO_3_, 2.3 KCl, 11 D-(+)-glucose, 1.0 NaH_2_PO_4_, 1.3 MgCl_2_ and 2.5 CaCl_2_ (pH 7.4, 300 mOsml^−1^).

The recording patch pipettes were filled with internal solution (in mmol/L): 128 K gluconate, 10 HEPES, 5 lidocaine N-ethyl chloride, 1.1 EGTA, 10 phosphocreatine Na salt, 5 ATP Mg salt, and 0.4 GTP Na salt (pH 7.35, 300 mOsml^−1^). The data were recorded with a multiclamp 700B amplifier (Molecular Devices). We chose a filter of 5 kHz and a sample rate of 20 kHz with a Digidata 1550B. We used Clampex 10.7 for acquisition and analysis. Neurons with a resting membrane potential more positive than −60 mV were discarded. Cells in which the series resistance (Rs, typically 8–12 MΩ) changed by >20% were excluded from the analysis. In addition, cells with Rs >20 MΩ at any time during the recordings were discarded.

1. **sEPSCs.** We recorded sEPSCs with the cell membrane potential held at −70 mV, 50 μmol/L APV, and 25 μmol/L bicuculline added to the ACSF.
2. **Paired-pulse Ratio (PPR).** The PPR was recorded while the cell membrane potential was at −70 mV. EPSCs were evoked with a bipolar platinum electrode placed in layers V/VI of the ACC and the recording pipette in layers II/III. We added 25 μmol/L bicuculline methobromide (BMR) and 50 μmol/L APV into the recording solution to block GABA transmission and NMDAR respectively. The inter-stimulus interval of the paired stimuli was 50 ms.
3. **AMPAR/NMDAR ratio.** We added 25 μmol/L BMR into the ACSF when the AMPAR- and NMDAR-mediated current ratio was recorded. AMPAR EPSCs were recorded as the peak current over a 2-ms window at −70 mV, while NMDAR EPSCs were recorded at +40 mV, with their magnitude measured at 50 ms after the onset of the EPSC. The ratio was calculated by dividing the AMPAR EPSC (measured as the peak amplitude at −70 mV) by the NMDAR EPSC (measured at 50 ms after the onset of EPSC at +40 mV).

### Statistical Analysis

All statistical analyses were conducted using Prism software, version 8.0 (GraphPad Software). Data are presented as the mean ± SEM. Specific details of the sample size for each study, such as the count of mice, slices, or neurons, can be found in the legends of the corresponding figures. Normality was assessed by the Shapiro-Wilk test. The homogeneity of variance test was assessed using Levene’s test. Normally distributed data of two conditions with equal variance were tested with a two-tailed unpaired Student's *t*-test. In scenarios where comparisons spanned more than two conditions within a single graphical representation, we opted for a one-way ANOVA, followed by a *post hoc* analysis using Tukey’s test to scrutinize all pairwise comparisons. For Sholl analysis, we used repeated-measures ANOVA. Datasets that were not normally distributed were analyzed with nonparametric tests. The significance of statistical findings was determined with a threshold of *P* <0.05 (‘n.s.’ no significant difference, *P <0.05, **P <0.01, ***P <0.001, and ****P <0.0001.

## Results

### Distribution Pattern of SHANK3 in the Adult Mouse Cerebral Cortex

**Fig. 1:**
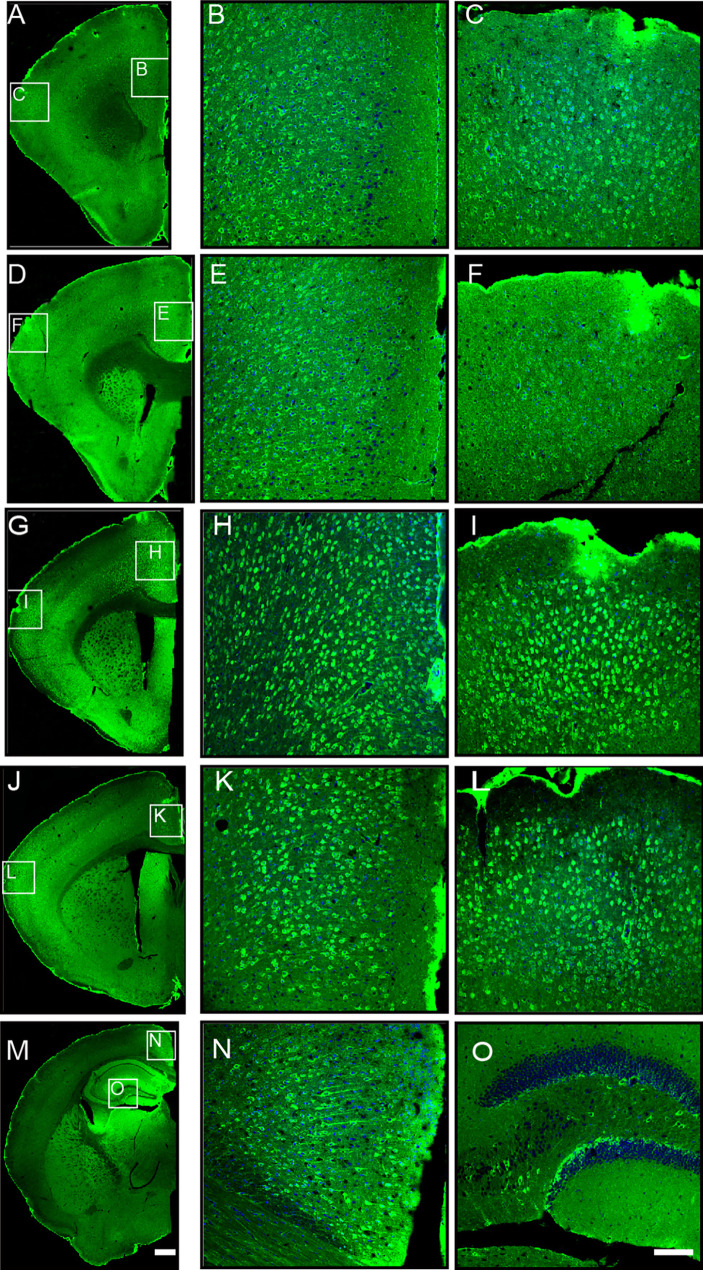
Distribution of SHANK3 immuno-positive cells in different regions of the adult mouse cerebral cortex. ** A, D, G, J, M** Whole-brain images showing the distribution of SHANK3-positive cells in different regions of the cerebral cortex in adult mice. **B, C** High-magnification images showing the distribution of SHANK3-positive cells in the prefrontal cortex (**B**) and S region (**C**) as shown in image **A**. **E**, **F** High-magnification views of SHANK3-positive cells in the ACC region (**E**) and S region (**F**) as shown in image **D**. **H**, **I** High-magnification views of SHANK3-positive cells in the ACC region (**H**) and S region (**I**) as shown in image **G**. **K**, **L** High-magnification views of SHANK3-positive cells in the ACC region (**K**) and S region (**L**) as shown in image **J. N**, **O** High-magnification views of SHANK3-positive cells in the RSPV region (**N**) and hippocampal region (**O**) as shown in image **M**. Green, SHANK3-positive cells; blue, cell nuclei. Scale bars, 300 μm (**A, D, G, J, M**); 100 μm (**B**. **C, E**, **F, H**, **I, K**, **L, N**, **O**).

Before delving into the role of *Shank3* across developmental stages, we first sought to objectively examine the expression patterns of SHANK3 in different cortical regions, determining the specific areas to focus on during development. We applied immunofluorescent staining with SHANK3-specific antibody that targets the SH3 domain to visualize its expression across various cortical regions. The specificity of this antibody was validated with the *Shank3B* KO mouse (sFig. 1 A). Coronal sections from adult mice were stained from the rostral to the caudal extent of the brain. The findings revealed a ubiquitous distribution of SHANK3-positive cells throughout the cortex, with differential expression levels across sections. Specifically, intense SHANK3 expression was noted from Bregma 0 to Bregma 0.98 mm, while moderate expression was found elsewhere (Fig. 1A, D, G, J, M). In addition, SHANK3-positive cell distribution varied among cortical subdivisions. Elevated expression levels were prominent in the ACC and somatosensory cortex, with relatively lower levels in other subcortical regions (Fig. 1B, C, E, F, H, I, K, L, N, O).

### Changes in SHANK3 Expression Levels and Distribution Patterns of SHANK3-Positive Cells in the Mouse Cerebral Cortex at Different Developmental Stages

**Fig.2:**
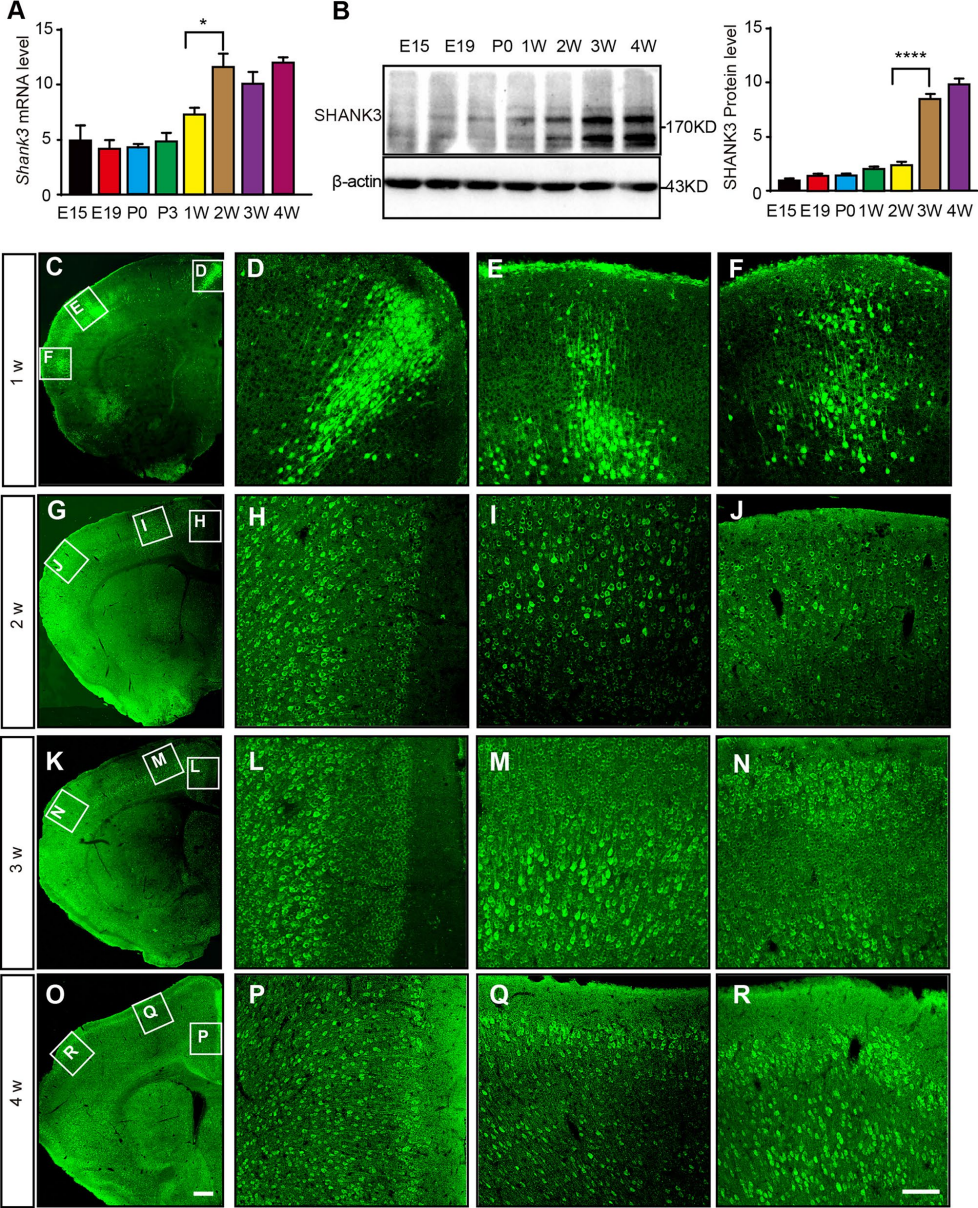
Expression levels and distribution patterns of SHANK3 at different developmental stages in the mouse cerebral cortex. **A** Changes in *Shank3* mRNA expression levels in the mouse cerebral cortex at different developmental stages (*n =* 3 mice/group, ^*^P <0.05 one-way ANOVA). **B** Western Blots and analysis of SHANK3 protein expression levels at different developmental stages (*n =* 3 mice/group, ^****^P <0.0001 one-way ANOVA). **C, G, K, O** Distribution of SHANK3 expression in the cerebral cortex of mice at 1, 2, 3, and 4 weeks after birth. Green, SHANK3-positive cells. W, weeks. **D–F** At 1-week post-birth, SHANK3-positive cells are mainly distributed in the M2 (**D**), S1 (**E**), and S2 (**F**) regions. **H–J** At 2 weeks after birth, SHANK3-positive cells are mainly distributed in the ACC (**H**), M1 (**I**), and S (**J**) regions. **L–N** At 3 weeks after birth, SHANK3-positive cells are mainly distributed in the ACC (**L**), M1 (**M**), and S (**N**) regions. **P–R** At 4 weeks after birth, SHANK3-positive cells are mainly distributed in the ACC (**P**), M (**Q**), and S2 (**R**) regions. Scale bars, 200μm (**C, G, K**, **O**); 100 μm (**D–F, H–J, L–N, P–R**). In this and subsequent figures, summary data are presented as the mean ± SEM.

To define the developmental time window in which *Shank3* acts, we initially assessed the expression level of SHANK3 at different time points. RT-PCR and Western Blot analyses were applied to track *Shank3* mRNA and protein expression changes during cortical development, respectively (sFig. 1B, C). *Shank3* mRNA was scarcely expressed prenatally, surged after the first postnatal week, and significantly escalated after the second week, subsequently plateauing (Fig. 2A). Corresponding protein levels remained low until a substantial increase from the third week onward (Fig. 2B).

Immunofluorescent staining charted the postnatal distribution of SHANK3-positive cells in the cortex. At one week, these cells were scant and confined mostly to the secondary motor cortex (M2) and S (Fig. 2D–F). By two weeks, their numbers and distribution expanded, concentrating in the ACC, primary motor cortex (M1), and S, particularly in deeper layers (Fig. 2H–J). At three weeks, a marked increase in SHANK3-positive cells occurred in the ACC, M1, and S regions, still favoring deeper layers (Fig. 2L–N). By four weeks, the distribution pattern mirrored that of adults, with high expression of SHANK3 in the ACC, M1, and S2, across both deep and superficial layers (Fig. 2P–R).

### Cytochemical Characteristics of SHANK3 in the Mouse ACC

**Fig. 3:**
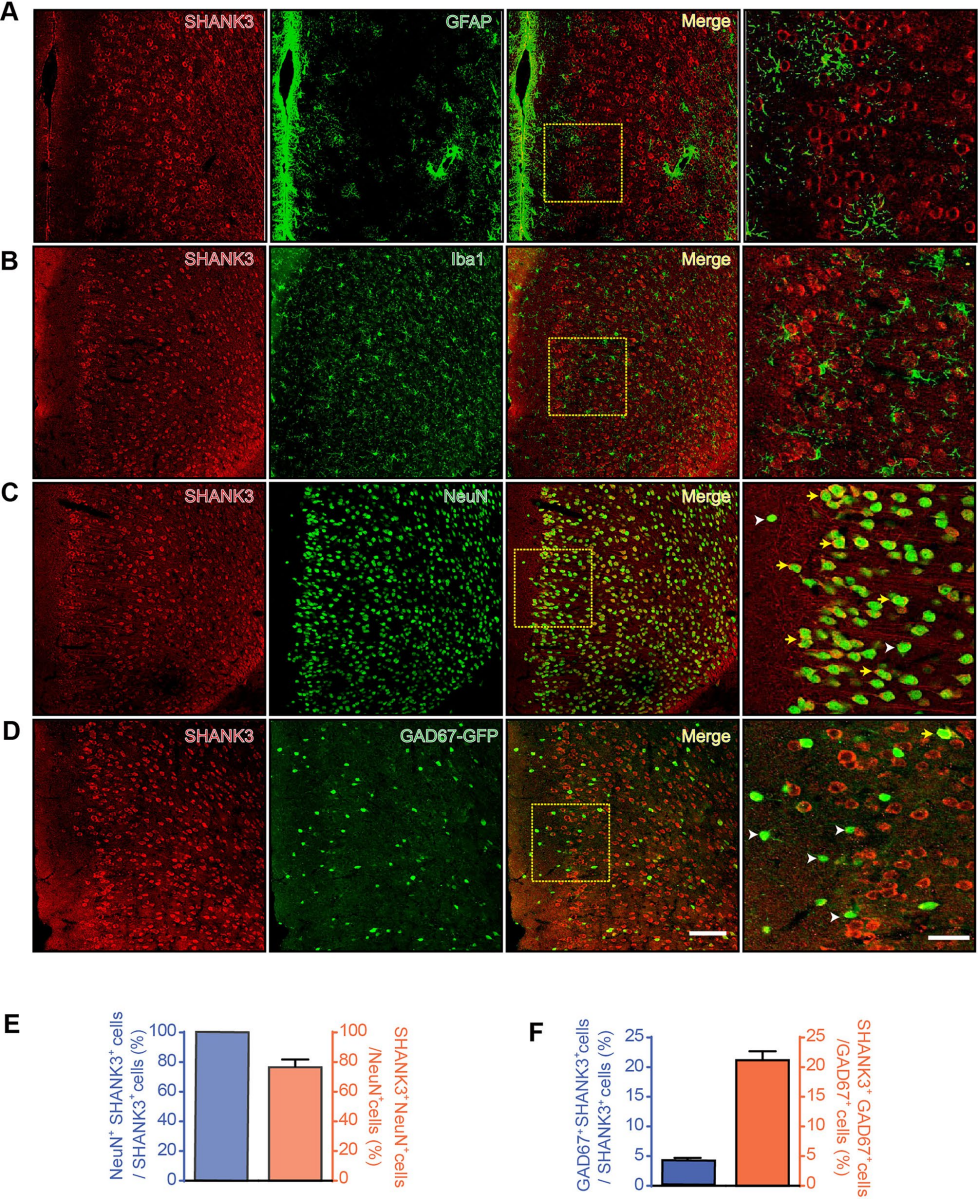
Double-immunostaining of SHANK3 with GFAP, Iba1, NeuN, GAD67 in the ACC of adult mice. **A** Double-immunostaining of SHANK3 and the astrocyte marker GFAP in the ACC. **B** Double-immunostaining of SHANK3 and the microglial marker Iba1 in the ACC. **C** Double-immunostaining of SHANK3 and the neuronal marker NeuN in the ACC. **D** Double-immunostaining of SHANK3 and the interneuron marker GAD67 in the ACC. The expression of GAD67 is visualized through green fluorescence in the GAD67-GFP mouse model. Figures in the last column are high-magnification views of the boxes in the third column. Yellow arrows, double-labeled cells; white arrowheads, non-double-labeled cells. Scale bars, 100 μm in the first three columns; 30 μm in the last column. **E, F** Summary data showing co-expression of SHANK3 and NeuN (**E**) and GAD67 (**F**). *n =* 6 slices from 3 mice/group.

As the high-expressing cortex in both adult and developing mice, the ACC was selected as the target region in which to examine the developmental effects of *Shank3*. First, we identified the cell types expressing SHANK3 using dual immunofluorescence labeling with NeuN (a neuronal marker), Iba1 (a microglial marker), and GFAP (an astrocytic marker). In the ACC, SHANK3-positive cells were predominantly found in layers II/III and V/VI, sparing layer I. Co-labeling demonstrated 77.1% ± 13.6% NeuN-positive cells expressed SHANK3 with all SHANK3-positive cells expressing NeuN (Fig. 3C, E). No co-labeling of SHANK3 with Iba1 or GFAP was detected (Fig. 3A, B), indicating exclusive neuronal expression of SHANK3. Further, our previously published *in situ* hybridization data showed SHANK3 expression in neurons also positive for the excitatory neuron marker αCaMKII [13]. Immunofluorescent staining here revealed that SHANK3 and the inhibitory neuron marker GAD67 rarely colocalized, with 4.1% ± 0.8% SHANK3-positive cells expressing GAD67 and 21.2% ± 4.2% GAD67-positive cells expressing SHANK3 (Fig. 3D, F), suggesting that SHANK3 is predominantly expressed in excitatory neurons.

### Impact of *Shank3* Knockout on Dendritic Morphology and Function of ACC Pyramidal Neurons in One-Week-Old Mice

**Fig. 4:**
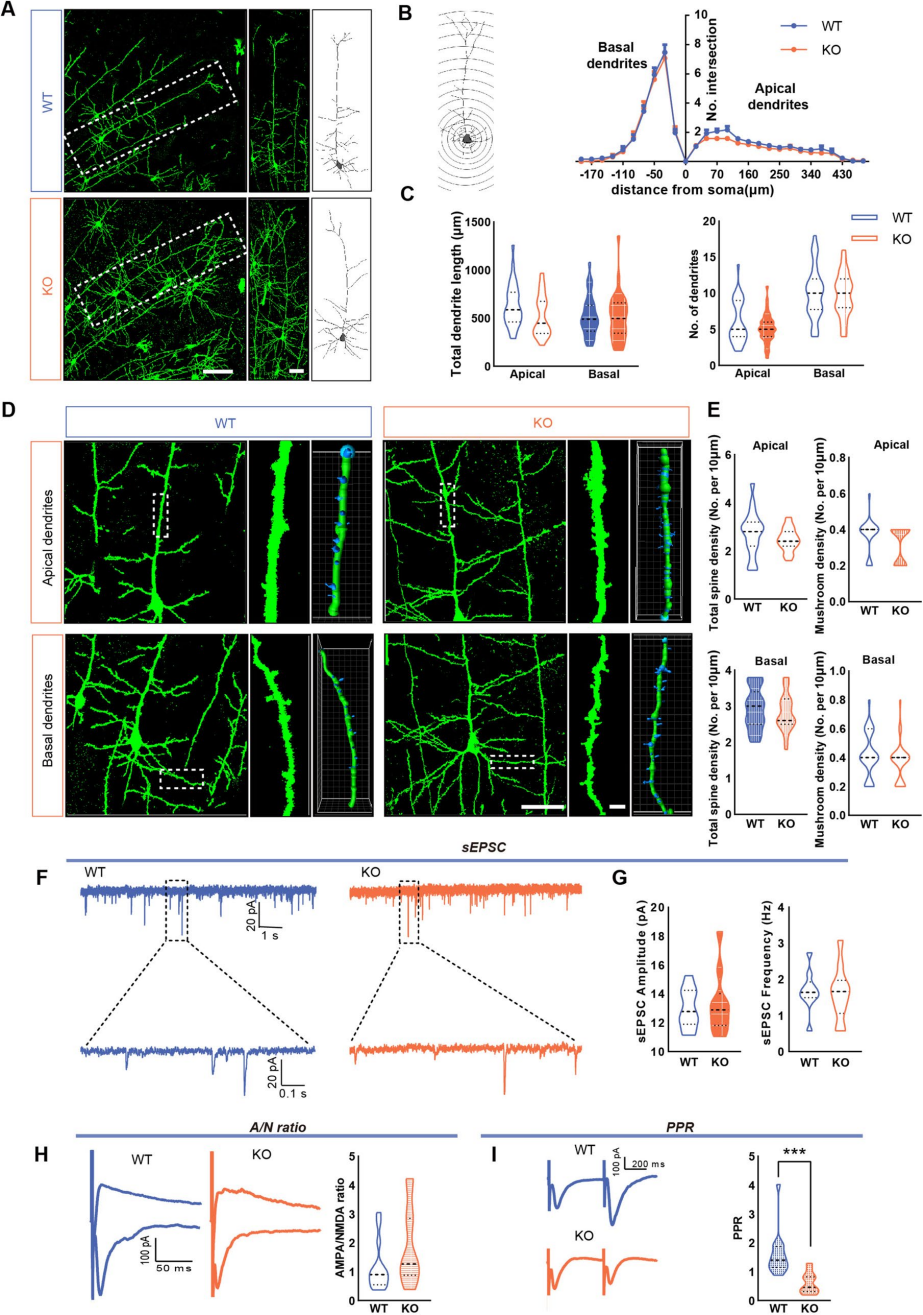
Impact of Shank3 knockout on the morphology and function of pyramidal neurons in the ACC of mice at 1 week after birth. **A** Typical Golgi staining images and redrawn images showing the morphology of pyramidal neurons in the ACC of WT and KO mice. Scale bars, 100 μm in the first column; 50 μm in the second column. **B** Sholl analysis of dendritic branching of pyramidal neurons in the ACC of WT and KO mice at different concentric radii (WT: *n =* 30 neurons from 3 mice, KO: *n =* 31 neurons from 3 mice, two-way ANOVA, repeated measures).** C** The total length of apical dendrites and basal dendrites and the total number of dendritic branches of pyramidal neurons in WT and KO mice (WT: *n =* 30 neurons from 3 mice, KO: *n =* 31 neurons from 3 mice, two-tailed unpaired t-test). **D** Typical Golgi staining images and Imaris software-reconstructed images showing the dendritic spine morphology of pyramidal neurons in the ACC of WT and KO mice. Scale bars, 50 μm in low magnification figures; 5 μm in high-magnification figures. **E** The density of dendritic spines on the apical and basal dendrites of pyramidal neurons (WT: *n =* 19 apical dendrites and *n =* 29 basal dendrites from 3 mice, KO: *n =* 20 apical dendrites and *n =* 29 basal dendrites from 3 mice, two-tailed unpaired *t-*test). **F** Representative sEPSC traces in ACC pyramidal neurons in WT and KO mice. **G** Summary data for sEPSC frequency and peak amplitude in ACC pyramidal neurons from WT and KO mice (WT: *n* = 10 neurons from 3 mice; KO: *n* = 10 neurons from 3 mice. Frequency: Mann–Whitney U-test; amplitude: two-tailed unpaired *t*-test). **H** Representative changes in AMPAR/NMDAR EPSC ratios of ACC pyramidal neurons in WT and KO mice and aggregated data (WT: *n* =14 neurons from 3 mice; KO: *n* =11 neurons from 3 mice. Mann–Whitney U-test). **I** Representative traces and statistical data of PPR in the two groups (WT: *n =* 14 neurons from 3 mice; KO: *n =* 11 neurons from 3 mice. ^***^P <0.001, two-tailed unpaired *t*-test).

To gain a deeper insight into the neurobiological impact of SHANK3 deficiency on neuronal morphology and synaptic functionality, we used a previously reported *Shank3B* KO mouse model [12]. The Golgi staining method was applied to assess the dendritic morphology of pyramidal neurons in the ACC of *Shank3B* KO mice at various developmental stages. At one week after birth, no significant differences in total dendrite length or branch number occurred between littermate wild-type (WT) and *Shank3B* KO mice (Fig. 4A, C). Sholl analysis also indicated no significant disparities in dendritic branching patterns (Fig. 4B). Three-dimensional dendritic spine analysis with Imaris software revealed no significant changes in apical and basal total dendritic spine density and mushroom-shaped spine density in *Shank3B* KO mice compared to the WT (Fig. 4D, E).

Whole-cell patch-clamp recordings were made to assess the impact of SHANK3 deficiency on synaptic transmission in ACC pyramidal neurons during development. To isolate excitatory synaptic activity, we incorporated 25 μmol/L BMR into the recording medium to inhibit GABA_A_ receptors. Our findings indicated no disparity in the frequency or peak amplitude of spontaneous excitatory postsynaptic currents (sEPSCs) between *Shank3B* KO and WT mice at this early developmental stage (Fig. 4F, G). In addition, to comprehensively evaluate both postsynaptic and presynaptic functions, we measured postsynaptic currents mediated by AMPA receptors (AMPARs) and NMDA receptors (NMDARs), as well as the paired-pulse ratio (PPR) as an index of presynaptic release. In terms of postsynaptic functionality, we analyzed the ratio of AMPAR to NMDAR currents and found no significant differences between WT and *Shank3B* KO mice (Fig. 4H). On the presynaptic side, however, we noted a reduced PPR in the *Shank3B* KO mice (Fig. 4I). Given the established role of SHANK3 in postsynaptic mechanisms, this increase in presynaptic release could potentially represent a compensatory response.

### Impact of *Shank3* Knockout on Dendritic Morphology and Synaptic Function of ACC Pyramidal Neurons in Two-Week-Old Mouse

**Fig. 5:**
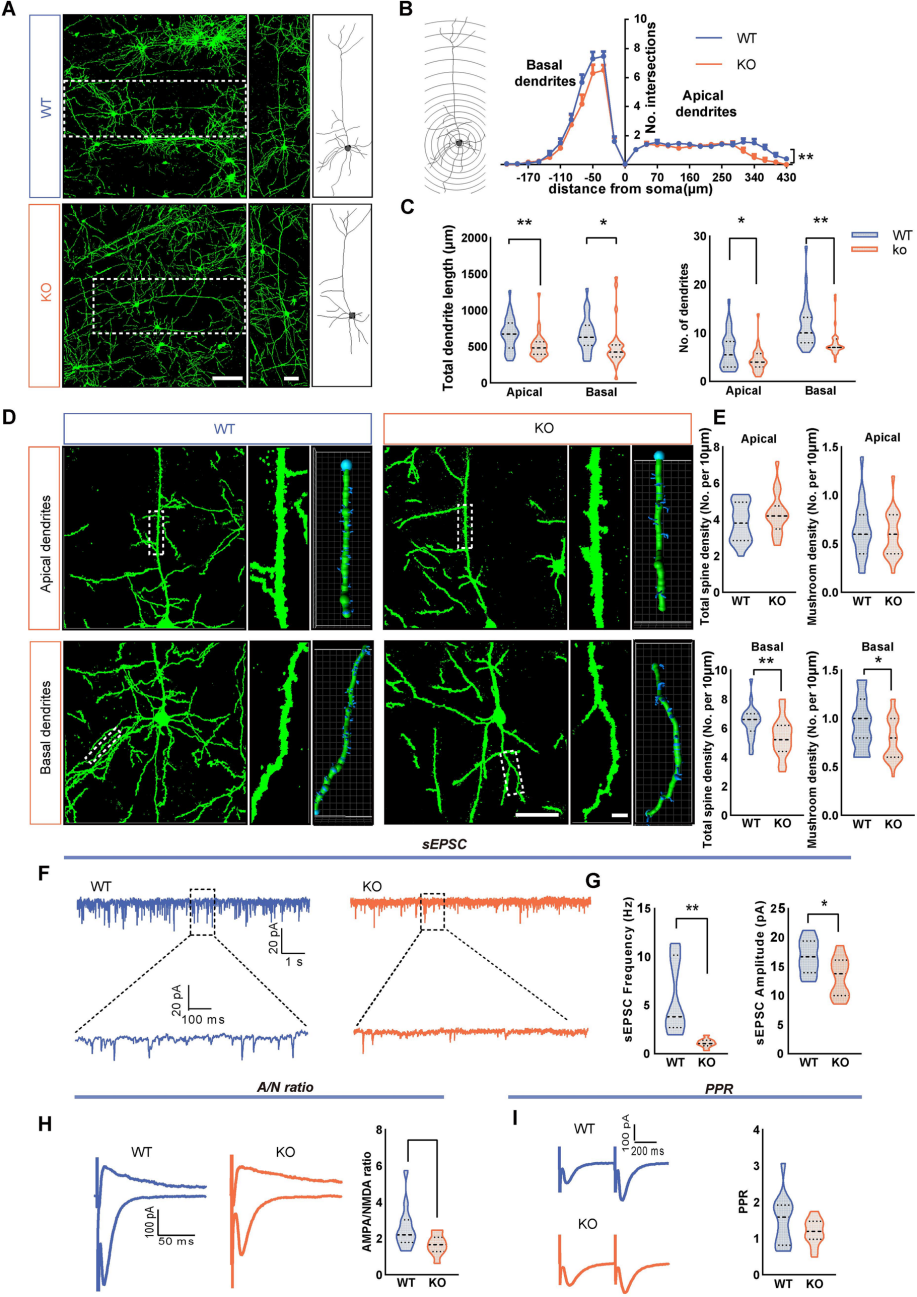
Impact of Shank3 knockout on the morphology and function of pyramidal neurons in the ACC of mice at 2 weeks after birth. **A** Typical Golgi-stained images and redrawn images of pyramidal neurons in the ACC of WT and KO mice. Scale bars, left panels 100 μm; right panels 50 μm. **B** Sholl analysis of dendritic branching of pyramidal neurons in the ACC of WT and KO mice at different concentric radii (WT: *n =* 30 neurons from 3 mice, KO: *n =* 28 neurons from 3 mice, ^**^P <0.01 two-way ANOVA repeated measures). **C** The total length of apical dendrites and basal dendrites and the total number of dendritic branches of pyramidal neurons in WT and KO mice (WT: *n =* 30 neurons from 3 mice, KO: *n =* 28 neurons from 3 mice, ^*^P <0.05, ^**^P <0.01 two-tailed unpaired t-test). **D** Typical Golgi staining images and Imaris software-reconstructed images showing the dendritic spine morphology of pyramidal neurons in the ACC of WT and KO mice. Scale bars, 50 μm in low-magnification figures; 5 μm in high-magnification figures. **E** The density of dendritic spines on apical and basal dendrites of pyramidal neurons (*n =* 20 apical and 23 basal dendrites from 3 mice/group, ^*^P <0.05, ^**^P <0.01 two-tailed unpaired t-test). **F** Representative sEPSC traces in ACC pyramidal neurons from WT and KO mice. **G** Compared with WT mice, the frequency and peak amplitude of ACC pyramidal neurons sEPSCs in KO mice are decreased (WT: *n* = 8 neurons from 3 mice; KO: *n* = 9 neurons from 3 mice. Frequency: ^**^P <0.01 Mann–Whitney U-test; amplitude: ^*^P <0.05 two-tailed unpaired *t*-test). **H** Representative changes in AMPAR/NMDAR ratios of ACC pyramidal neurons in WT and KO mice and aggregated data (WT: *n* =13 neurons from 3 mice; KO: *n* =11 neurons from 3 mice. ^*^P <0.05 Mann–Whitney U-test). **I** Representative traces and statistical data of PPR in the two groups (WT: *n =* 13 neurons from 3 mice; KO: *n =* 11 neurons from 3 mice. Two-tailed unpaired *t*-test).

At two weeks after birth, *Shank3B* KO mice exhibited significantly shorter apical and basal dendrites than the WT, along with fewer branches (Fig. 5A, C). Sholl analysis confirmed fewer intersection points in the apical dendrites of KO mice (Fig. 5B). Dendritic spine analysis showed no change in apical total spine density and mushroom-shaped spine density but a significant reduction in total basal spine density and mushroom spine density in *Shank3B* KO mice (Fig. 5D, E).

Despite morphological evidence of alterations in dendritic architecture and spine number in *Shank3B* KO mice two weeks postnatal, the corresponding synaptic functional deficits remained uncharacterized. To elucidate the effects of *Shank3* deletion on synaptic transmission in ACC pyramidal neurons, we measured the frequency and amplitude of sEPSCs. Our findings showed a reduction in both parameters in the *Shank3B* KO group (Fig. 5F, G). Subsequent measurements of AMPAR/NMDAR ratios revealed a decreased ratio in *Shank3B* KO mice compared to the WT (Fig. 5H), indicating a relative reduction in AMPA receptor components in the postsynaptic regions associated with SHANK3 deficiency. No significant alterations were detected in the PPR (Fig. 5I), suggesting that the presynaptic release probability was transiently altered at one week postnatal but returned to baseline by the second postnatal week.

### Impact of *Shank3* Gene Knockout on Dendritic Morphology and Synaptic Function of ACC Pyramidal Neurons in Three-Week-Old Mouse

**Fig. 6:**
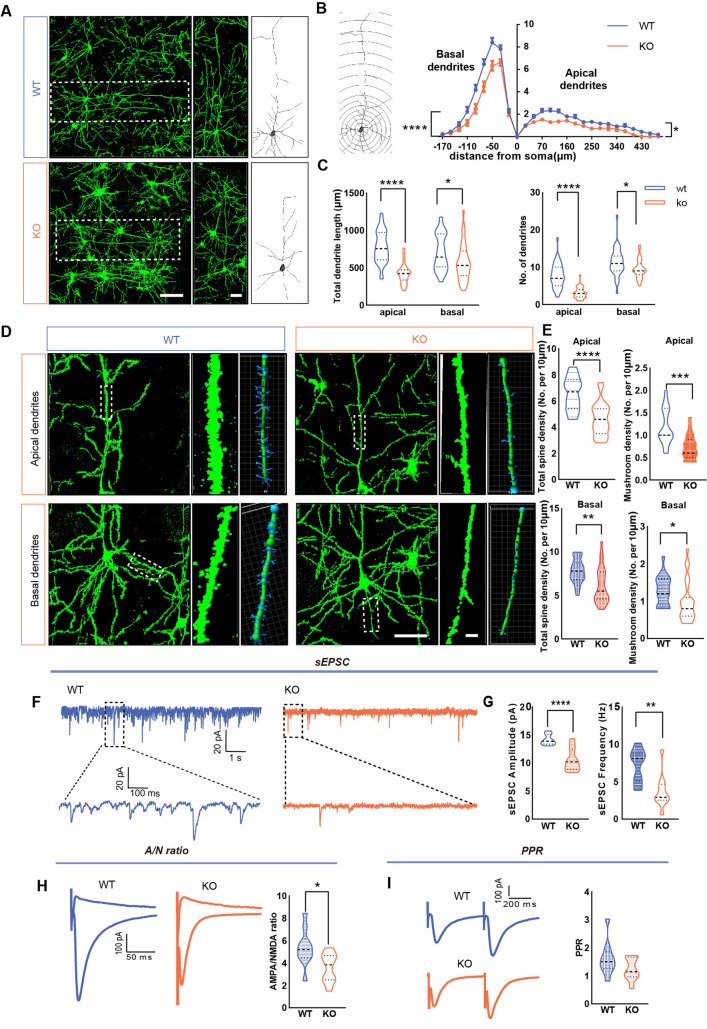
Impact of Shank3 knockout on the morphology and function of pyramidal neurons in the ACC of mice at 3 weeks after birth. **A** Typical Golg-stained images and redrawn images of pyramidal neurons in the ACC of WT and KO mice. Scale bars, left panels, 100 μm; right panels, 50 μm. **B** Sholl analysis of dendritic branching of pyramidal neurons in the ACC of WT and KO mice at different concentric radii (WT: *n =* 35 neurons from 3 mice, KO: *n =* 31 neurons from 3 mice, ^*^P <0.05, ^****^P <0.0001, two-way ANOVA repeated measures). **C** The total length of apical and basal dendrites and the total number of dendritic branches of pyramidal neurons in WT and KO mice (WT: *n =* 35 neurons from 3 mice, KO: *n =* 31 neurons from 3 mice, ^*^P <0.05, ^****^P <0.0001 two-tailed unpaired *t*-test). **D** Typical Golgi-stained images and Imaris software-reconstructed images showing the dendritic spine morphology of pyramidal neurons in the ACC of WT and KO mice. Scale bars, 50 μm in low-magnification figures; 5 μm in high-magnification figures. **E** The density of dendritic spines on the apical and basal dendrites of pyramidal neurons (WT: *n =* 20 apical and 21 basal dendrites from 3 mice, KO: *n =* 21 apical and 26 basal dendrites from 3 mice. ^*^P <0.05, ^**^P <0.01, ^***^P <0.001, ^****^P <0.0001 two-tailed unpaired *t*-test). **F** Representative sEPSC traces in ACC pyramidal neurons in WT and KO mice. **G** Compared with WT mice, the frequency and peak amplitude of ACC pyramidal neuronal sEPSCs in KO mice are decreased. (WT: *n* = 10 neurons from 3 mice; KO: *n* = 10 neurons from 3 mice. Frequency: ^**^P <0.01 Mann–Whitney U-test; amplitude: ^****^P <0.0001 two-tailed unpaired *t*-test). **H** Representative traces and data summary of AMPAR/NMDAR ratios in pyramidal neurons of the ACC, with lower AMPAR/NMDAR ratios in KO mice than in WT mice (WT: *n* = 10 neurons from 3 mice; KO: *n* = 9 neurons from 3 mice. ^*^P <0.05 Mann–Whitney U-test). **I** Representative traces and statistical data of PPR in the two groups (WT: *n =* 10 neurons from 3 mice; KO: *n =* 9 neurons from 3 mice. Two-tailed unpaired *t*-test).

By three weeks, *Shank3B* KO mice displayed a drastic reduction in both the length and branching of apical and basal dendrites compared to the WT (Fig. 6A, C). Sholl analysis revealed a significant reduction in intersection points of basal dendrites and apical dendrites in KO mice (Fig. 6B). Furthermore, there was a significant reduction in the density of total dendritic spines and mushroom-shaped spines on both apical and basal dendrites in the *Shank3B* KO group (Fig. 6D, E).

We investigated whether *Shank3B* KO continued to affect synaptic function at this critical period for neuronal maturation. Persistent reductions in the frequency and amplitude of sEPSCs were found in *Shank3B* KO mice (Fig. 6F, G). Analysis of the AMPAR/NMDAR ratio further supported these findings, with *Shank3B* KO mice displaying lower ratios than their WT counterparts (Fig. 6H). Yet, PPR remained unchanged between the groups (Fig. 6I).

## Discussion

In this study, we assessed the expression levels and patterns of SHANK3 in the murine cerebral cortex during the first three postnatal weeks and explored the impact of SHANK3 deficiency on the morphological and synaptic development of pyramidal neurons in the ACC at various developmental stages using *Shank3B* KO mice. Our findings showed that SHANK3 is robustly expressed in the pyramidal neurons within the ACC, M, and S regions of the mouse cerebral cortex during early postnatal stages. SHANK3 deficiency resulted in impaired dendrite formation and synaptic transmission, underscoring the protein's critical role in early neuronal development.

SHANK3, a member of the family of excitatory postsynaptic density proteins, is widespread in the brain, with pronounced expression in the cerebral cortex [12, 26]. Applying immunofluorescence staining, we discerned SHANK3 protein expression levels across different regions of the cerebral cortex. Elevated SHANK3 expression was detected in the ACC and S (mainly S2) regions in the adult mouse cortex, while other regions manifested comparatively lower expression levels. This expression pattern implicates *Shank3* in the functional integrity of these brain regions. Previous research has shown that the ACC is engaged in various executive functions, including error and conflict monitoring [27], social behavior [28], decision-making [29], hyperalgesia, and anxiety [30]. Further, our prior investigations in adult mice have established a direct association between ACC abnormalities and social behavior deficits in *Shank3* mutant mice [13]. The somatosensory cortex, which processes information from primary sensory systems and is crucial for sensory integration, including vision, hearing, taste, and proprioception, has been implicated in autism, as ~90% of individuals with autism exhibit deficits in sensory perception [31]. A previous study has built the causal link between the hyperactivity of the somatosensory cortex and hypersensitivity in *Shank3B* KO mice [32]. While different cell types populate the cortex, our study revealed that SHANK3 is predominantly expressed in excitatory glutamatergic neurons, with a minor population of inhibitory neurons expressing SHANK3 and an absence of expression in glial cells. Our current and previous studies have concentrated on *Shank3*'s role in excitatory glutamatergic neurons; however, previous research has demonstrated that selective disruption of *Shank3* in cortical interneurons can lead to cortical hyperactivity [32]. These findings highlight the necessity of investigating the functions of *Shank3* across various neuronal subtypes to elucidate the mechanisms underlying ASD.

To ascertain if the adult expression pattern of SHANK3 has its origins in developmental stages, we examined SHANK3 protein expression and distribution during neuronal development. Previous studies have shown that many *Shank3* isoforms are produced in the developing mouse brain [9, 33–35]. In our study, we used *Shank3* primers that target exons 5–8 and discovered that *Shank3* mRNA levels, which may reflect the expression of *Shank3a* and *Shank3b*, increased after the first week after birth, with a pronounced spike in the cerebral cortex at two weeks. This result aligns somewhat with the findings of Wang *et al*. [33], who reported a surge in *Shank3a* mRNA levels in the mouse brain at postnatal day 10, followed by a plateau until four weeks. Applying western blot analysis with an antibody specific to the SH3 domain of SHANK3, which theoretically detects the expression of *Shank3a, Shank3b,* and *Shank3c*, we examined the protein expression in the cerebral cortex. The results showed that the protein levels were subdued at one and two weeks but increased significantly by the third week. Further investigation using immunofluorescence with the same antibody to assess protein distribution in the cortex revealed that SHANK3 expression was initially confined to the M2, parts of S1, and the S2 regions one week after birth, but began to expand from the second week onwards. The changing expression and distribution patterns of SHANK3 during development imply its involvement in stage-specific neurological functions, potentially reflecting the varied behavioral symptoms seen in autistic individuals at different ages. Previous studies have documented that autistic children often experience developmental delays in fundamental motor skills, such as sitting, standing, and walking, before the age of one [36]. This is followed by significant language deficits, social interaction difficulties, and the emergence of repetitive behaviors [37]. These findings align with our finding that SHANK3 is initially restricted to the M2 region shortly after birth and subsequently extends to other areas. Moreover, the early behavioral anomalies in *Shank3B* knockout mice—such as reduced total distance traveled and rearing from P15, less time in the central arena at P15, deficient performance on the rotarod at P17-P20, and increased grooming during P15–P20—coincide with the timeline of *Shank3* expression, which intensifies from the second postnatal week. This temporal correlation further supports a potential link between SHANK3 expression patterns and the onset of behavioral symptoms [38]. Consequently, the role of the differential expression of *Shank3* in the pathogenesis of ASD warrants in-depth exploration to elucidate its contribution to the disorder's progression.

The postnatal period is pivotal for the morphological development of neurons in the cerebral cortex, encompassing migration, polarization, axon and dendrite formation and pruning, dendritic spine development, and synaptogenesis—all tightly regulated by intrinsic and extrinsic factors [39, 40]. In the initial week after birth, immature neurons predominantly complete migration and polarization, with sparse dendritic spine formation, most of which are not functional [41]. We found that at one week after birth, SHANK3 expression is initially low, suggesting a limited role in the early stages. Through Golgi staining of pyramidal neurons in the ACC, we ascertained that *Shank3* gene knockout did not substantially alter dendritic length or branching. However, as expression increases, its influence on dendritic development becomes evident. SHANK3 deficiency reduces dendritic length, total spine density, and mushroom-shaped spine density, particularly by the third week, indicating its importance in dendritic maturation. The ramifications of these morphological alterations due to SHANK3 deficiency are considerable. MRI studies have revealed disorganized brain connectivity patterns in autistic individuals, typified by reduced large-scale and elevated small-scale connections [42, 43], yet the cellular underpinnings of these abnormalities remain elusive. While dendrites have garnered attention for their critical role in cellular connectivity, the focus has predominantly been on dendritic spine and synapse formation [44, 45]. However, these may not be the sole cellular contributors. Some studies have linked macrocephaly or microcephaly in certain autistic children to aberrant dendritic branching during development [46–49]. Our research posits that the developmental alterations in pyramidal neuron dendrites resulting from *Shank3* gene knockout may contribute to the etiology of ASD.

Bozdagi *et al.* [17] reported that adult mice with hemizygous SHANK3 deficiency exhibited reduced mEPSC amplitude in hippocampal CA1 pyramidal neurons. Similarly, Peca *et al.* [12] reported significant reductions in AMPA receptor-mediated mEPSC amplitude in medium spiny neurons (MSN) cells in the dorsolateral striatum of *Shank3B* KO mice. These studies suggest that synaptic alterations are accompanied by changes in neuronal morphology. Our experimental work indicates that during development, SHANK3 protein modulates dendritic branching and spine density in cerebral cortex pyramidal neurons, prompting the question: do these morphological changes coincide with functional alterations in neurons? Our findings indicate that at one week after birth, *Shank3* knockout only increases the presynaptic release probability in ACC pyramidal neurons. However, at two and three weeks, *Shank3* knockout significantly reduces both sEPSC frequency and amplitude. sEPSCs reflect the spontaneous activity of excitatory synapses in the absence of external stimuli, with frequency indicative of presynaptic neurotransmitter release and amplitude changes reflective of postsynaptic receptor properties [50]. During the first postnatal week, the reduction in postsynaptic functionality may not yet be pronounced, allowing synapses to maintain their overall function through increased presynaptic release, hence the changes in PPR. This increase in presynaptic release could be a compensatory mechanism to counterbalance initial postsynaptic deficits. However, by the second week and beyond, the postsynaptic alterations become more severe and important, leading to a decompensation of synaptic function. As a result, the compensatory presynaptic changes in the first week are no longer evident, resulting in no significant changes in PPR at later stages. This temporally dependent functionality is further corroborated by a previous study, which demonstrated that striatal spiny projection neurons in *Shank3B* KO mice exhibit an increased mEPSC frequency from the second postnatal week, which is similar to our finding in the first week after birth [38]. On the other hand, previous studies have shown that SHANK3 may also play important roles in the axon and presynaptic area [51]. The variability in impact across different developmental stages is presumably attributable to the differential expression levels of SHANK3 during developmental stages. It is possible that SHANK3 plays a more important presynaptic role during the first postnatal week, leading to the evident presynaptic changes in KO mice in postnatal week 1. Further experiments are necessary to investigate this intriguing issue regarding why *Shank3* deletion may lead to distinct synaptic alterations at different postnatal stages.

Ionotropic glutamate receptors play integral roles in synaptic transmission [52, 53]. During cortical development, the ratio of these receptors on the membrane evolves [54–57]. In early postnatal cortical development, NMDA receptor prevalence is higher on the postsynaptic membrane, yet these receptors do not generate significant currents at resting potentials, resulting in silent synapses [58]. As development proceeds, the silent synapses recruit AMPA receptors in an activity-dependent manner, becoming predominant in mature synapses [56, 59–62]. In this developmental trajectory, SHANK3, the postsynaptic scaffold protein that traffics NMDA and AMPA receptors, has been shown to increase its synaptic localization alongside the GluA2 subunit-containing AMPA receptors [63]. Moreover, the downregulation of SHANK3 is associated with the enhanced insertion of GluA2-lacking AMPARs on DA neurons [64], underscoring the critical role of SHANK3 in the maturation of AMPARs. Our investigation has revealed that the knockout of *Shank3* affects the functional ratio of AMPA and NMDA receptors in the postsynaptic compartment. The surface trafficking of AMPARs represents a vulnerable pathway in autism [65]. Consistent with previous findings from postmortem brain tissue analyses of individuals with autism, there is evidence of reduced AMPAR density in the cerebellum of these patients [66]. Furthermore, a clinical trial administering the AMPAR-positive allosteric modulator piracetam to 40 autistic children, in conjunction with risperidone, resulted in higher behavioral scores than in those treated with risperidone alone [67]. This finding implies that a combined therapeutic approach involving antipsychotic medications and glutamate receptor modulators may yield more promising results in the treatment of autism.

To decipher the developmental role of SHANK3 in dendritic morphology and synaptic function within the cerebral cortex, this study primarily focused on the ACC. We examined the morphological and electrophysiological changes in neurons within this specific brain region. While our findings provide insights into the role of SHANK3 in the ACC, they may not be fully representative of changes across all brain regions. It is also possible that the impact of SHANK3 deficiency extends beyond the ACC, and the observed alterations may not capture the full spectrum of effects that occur in other areas of the brain. Future studies that encompass a broader range of brain regions will be essential to comprehensively understand the neurodevelopmental implications of SHANK3 deficiency. Furthermore, in this study, we utilized a *Shank3B* KO mouse model, which resulted in the absence of expression of the alpha and beta isoforms of *Shank3*—recently referred to as *Shank3a* and *Shank3b*—and a reduced expression of the gamma isoform, which has been more recently designated as *Shank3c* or *Shank3d*. This model, therefore, does not fully represent the functional spectrum of all *Shank3* isoforms. The morphological and functional alterations found in the *Shank3B* KO mice are indicative of the specific roles played by the disrupted isoforms, rather than a comprehensive understanding of the overall function of *Shank3*. This distinction is crucial when interpreting the results and drawing conclusions about the broader biological impact of *Shank3*. To further elucidate the specific contributions of each isoform, additional research is warranted.

In summary, our study delineates the distribution of SHANK3 protein in the adult and developing murine cerebral cortex, with pronounced expression in the ACC, M, and S regions, and an age-related increase in expression postnatally, primarily in pyramidal neurons. By examining the morphology and synaptic function of ACC pyramidal neurons at different developmental stages in *Shank3B* KO mice, we have demonstrated *Shank3*'s role in dendrite formation and synaptic function *in vivo*. Our research adds to the growing body of evidence linking *Shank3* to the neurobiological underpinnings of ASD. By dissecting the developmental trajectory of SHANK3 expression and its impact on neuronal and synaptic development, we provide insights into the cellular mechanisms that may underlie the clinical manifestations of ASD. Furthermore, our results, which harmonize the spatiotemporal expression pattern of SHANK3 with the morphological and functional alterations post-knockout, are congruent with the behavioral insights reported by Peixoto *et al*. [38]. This concordance points to a compelling avenue for future research. Specifically, the temporal modulation of SHANK3 re-expression could uncover critical periods for intervention, offering prospects to reverse or mitigate ASD-like phenotypes. The advent of the *Shank3*^fx/fx^ mouse model [68] now paves the way for such investigations, enabling the exploration of re-expression's impact on circuit-level functionality and behavior. This approach holds promise for uncovering the therapeutic potential of modulating SHANK3 pathways in ASD.

## Supplementary Information

Below is the link to the electronic supplementary material.

Supplementary file1 (PDF 370 KB)

## Data Availability

The datasets used in this study are available from the corresponding author upon reasonable request.

## References

[1] Lai MC, Lombardo MV, Baron-Cohen S. Autism. Lancet 2014, 383: 896–910. 24074734 10.1016/S0140-6736(13)61539-1

[2] Hirota T, King BH. Autism spectrum disorder: A review. JAMA 2023, 329: 157–168. 36625807 10.1001/jama.2022.23661

[3] Höfer J, Hoffmann F, Kamp-Becker I, Poustka L, Roessner V, Stroth S. Pathways to a diagnosis of autism spectrum disorder in Germany: A survey of parents. Child Adolesc Psychiatry Ment Health 2019, 13: 16. 30949235 10.1186/s13034-019-0276-1 PMC6429704

[4] De Rubeis S, Buxbaum JD. Genetics and genomics of autism spectrum disorder: Embracing complexity. Hum Mol Genet 2015, 24: R24–R31. 26188008 10.1093/hmg/ddv273 PMC4675826

[5] Sanders SJ, Xin H, Willsey AJ, Ercan-Sencicek AG, Samocha KE, Cicek AE, *et al*. Insights into autism spectrum disorder genomic architecture and biology from 71 risk loci. Neuron 2015, 87: 1215–1233. 26402605 10.1016/j.neuron.2015.09.016 PMC4624267

[6] Durand CM, Betancur C, Boeckers TM, Bockmann J, Chaste P, Fauchereau F, *et al*. Mutations in the gene encoding the synaptic scaffolding protein SHANK3 are associated with autism spectrum disorders. Nat Genet 2007, 39: 25–27. 17173049 10.1038/ng1933 PMC2082049

[7] Moessner R, Marshall CR, Sutcliffe JS, Skaug J, Pinto D, Vincent J, *et al*. Contribution of SHANK3 mutations to autism spectrum disorder. Am J Hum Genet 2007, 81: 1289–1297. 17999366 10.1086/522590 PMC2276348

[8] Gauthier J, Spiegelman D, Piton A, Lafrenière RG, Laurent S, St-Onge J, *et al*. Novel *de novo* SHANK3 mutation in autistic patients. Am J Med Genet Part B Neuropsychiatr Genet 2009, 150B: 421–424. 10.1002/ajmg.b.30822 18615476

[9] Wang X, McCoy PA, Rodriguiz RM, Pan Y, Je HS, Roberts AC, *et al*. Synaptic dysfunction and abnormal behaviors in mice lacking major isoforms of Shank3. Hum Mol Genet 2011, 20: 3093–3108. 21558424 10.1093/hmg/ddr212 PMC3131048

[10] Kouser M, Speed HE, Dewey CM, Reimers JM, Widman AJ, Gupta N, *et al*. Loss of predominant Shank3 isoforms results in hippocampus-dependent impairments in behavior and synaptic transmission. J Neurosci 2013, 33: 18448–18468. 24259569 10.1523/JNEUROSCI.3017-13.2013 PMC3834052

[11] Lee J, Chung C, Ha S, Lee D, Kim DY, Kim H, *et al*. Shank3-mutant mice lacking exon 9 show altered excitation/inhibition balance, enhanced rearing, and spatial memory deficit. Front Cell Neurosci 2015, 9: 94. 25852484 10.3389/fncel.2015.00094 PMC4365696

[12] Peça J, Feliciano C, Ting JT, Wang W, Wells MF, Venkatraman TN, *et al*. Shank3 mutant mice display autistic-like behaviours and striatal dysfunction. Nature 2011, 472: 437–442. 21423165 10.1038/nature09965 PMC3090611

[13] Guo B, Chen J, Chen Q, Ren K, Feng D, Mao H, *et al*. Anterior cingulate cortex dysfunction underlies social deficits in Shank3 mutant mice. Nat Neurosci 2019, 22: 1223–1234. 31332372 10.1038/s41593-019-0445-9

[14] Naisbitt S, Kim E, Tu JC, Xiao B, Sala C, Valtschanoff J, *et al*. Shank, a novel family of postsynaptic density proteins that binds to the NMDA receptor/PSD-95/GKAP complex and cortactin. Neuron 1999, 23: 569–582. 10433268 10.1016/s0896-6273(00)80809-0

[15] Sheng M, Kim E. The Shank family of scaffold proteins. J Cell Sci 2000, 113(Pt 11): 1851–1856. 10806096 10.1242/jcs.113.11.1851

[16] Monteiro P, Feng G. SHANK proteins: Roles at the synapse and in autism spectrum disorder. Nat Rev Neurosci 2017, 18: 147–157. 28179641 10.1038/nrn.2016.183

[17] Bozdagi O, Sakurai T, Papapetrou D, Wang X, Dickstein DL, Takahashi N, *et al*. Haploinsufficiency of the autism-associated Shank3 gene leads to deficits in synaptic function, social interaction, and social communication. Mol Autism 2010, 1: 15. 21167025 10.1186/2040-2392-1-15 PMC3019144

[18] Zhu Y, Sousa AMM, Gao T, Skarica M, Li M, Santpere G, *et al*. Spatiotemporal transcriptomic divergence across human and macaque brain development. Science 2018, 362: eaat8077. 30545855 10.1126/science.aat8077 PMC6900982

[19] Roussignol G, Ango F, Romorini S, Tu JC, Sala C, Worley PF, *et al*. Shank expression is sufficient to induce functional dendritic spine synapses in aspiny neurons. J Neurosci 2005, 25: 3560–3570. 15814786 10.1523/JNEUROSCI.4354-04.2005 PMC6725374

[20] Verpelli C, Dvoretskova E, Vicidomini C, Rossi F, Chiappalone M, Schoen M, *et al*. Importance of Shank3 protein in regulating metabotropic glutamate receptor 5 (mGluR5) expression and signaling at synapses. J Biol Chem 2011, 286: 34839–34850. 21795692 10.1074/jbc.M111.258384 PMC3186429

[21] Yi F, Danko T, Botelho SC, Patzke C, Pak C, Wernig M, *et al*. Autism-associated SHANK3 haploinsufficiency causes Ih channelopathy in human neurons. Science 2016, 352: aaf2669. 26966193 10.1126/science.aaf2669 PMC4901875

[22] Wang Y, Chiola S, Yang G, Russell C, Armstrong CJ, Wu Y, *et al*. Modeling human telencephalic development and autism-associated SHANK3 deficiency using organoids generated from single neural rosettes. Nat Commun 2022, 13: 5688. 36202854 10.1038/s41467-022-33364-z PMC9537523

[23] Tamamaki N, Yanagawa Y, Tomioka R, Miyazaki JI, Obata K, Kaneko T. Green fluorescent protein expression and colocalization with calretinin, parvalbumin, and somatostatin in the GAD67-GFP knock-in mouse. J Comp Neurol 2003, 467: 60–79. 14574680 10.1002/cne.10905

[24] He X, Li J, Zhou G, Yang J, McKenzie S, Li Y, *et al*. Gating of hippocampal rhythms and memory by synaptic plasticity in inhibitory interneurons. Neuron 2021, 109: 1013-1028.e9. 33548174 10.1016/j.neuron.2021.01.014 PMC9239733

[25] Guo B, Xi K, Mao H, Ren K, Xiao H, Hartley ND, *et al*. CB1R dysfunction of inhibitory synapses in the ACC drives chronic social isolation stress-induced social impairments in male mice. Neuron 2024, 112: 441–457.e6. 37992714 10.1016/j.neuron.2023.10.027

[26] Boeckers TM, Winter C, Smalla KH, Kreutz MR, Bockmann J, Seidenbecher C, *et al*. Proline-rich synapse-associated proteins ProSAP1 and ProSAP2 interact with synaptic proteins of the SAPAP/GKAP family. Biochem Biophys Res Commun 1999, 264: 247–252. 10527873 10.1006/bbrc.1999.1489

[27] Botvinick MM, Cohen JD, Carter CS. Conflict monitoring and anterior cingulate cortex: An update. Trends Cogn Sci 2004, 8: 539–546. 15556023 10.1016/j.tics.2004.10.003

[28] Etkin A, Egner T, Kalisch R. Emotional processing in anterior cingulate and medial prefrontal cortex. Trends Cogn Sci 2011, 15: 85–93. 21167765 10.1016/j.tics.2010.11.004 PMC3035157

[29] Haber SN, Behrens TEJ. The neural network underlying incentive-based learning: Implications for interpreting circuit disruptions in psychiatric disorders. Neuron 2014, 83: 1019–1039. 25189208 10.1016/j.neuron.2014.08.031 PMC4255982

[30] Ren D, Li JN, Qiu XT, Wan FP, Wu ZY, Fan BY, *et al*. Anterior cingulate cortex mediates hyperalgesia and anxiety induced by chronic pancreatitis in rats. Neurosci Bull 2022, 38: 342–358. 34907496 10.1007/s12264-021-00800-x PMC9068840

[31] Robertson CE, Baron-Cohen S. Sensory perception in autism. Nat Rev Neurosci 2017, 18: 671–684. 28951611 10.1038/nrn.2017.112

[32] Chen Q, Deister CA, Gao X, Guo B, Lynn-Jones T, Chen N, *et al*. Dysfunction of cortical GABAergic neurons leads to sensory hyper-reactivity in a Shank3 mouse model of ASD. Nat Neurosci 2020, 23: 520–532. 32123378 10.1038/s41593-020-0598-6 PMC7131894

[33] Wang X, Xu Q, Bey AL, Lee Y, Jiang YH. Transcriptional and functional complexity of Shank3 provides a molecular framework to understand the phenotypic heterogeneity of SHANK3 causing autism and Shank3 mutant mice. Mol Autism 2014, 5: 30. 25071925 10.1186/2040-2392-5-30 PMC4113141

[34] Waga C, Asano H, Sanagi T, Suzuki E, Nakamura Y, Tsuchiya A, *et al*. Identification of two novel Shank3 transcripts in the developing mouse neocortex. J Neurochem 2014, 128: 280–293. 24164323 10.1111/jnc.12505

[35] Okuzono S, Fujii F, Matsushita Y, Setoyama D, Shinmyo Y, Taira R, *et al*. Shank3a/b isoforms regulate the susceptibility to seizures and thalamocortical development in the early postnatal period of mice. Neurosci Res 2023, 193: 13–19. 36871873 10.1016/j.neures.2023.03.001

[36] Arabameri E, Sotoodeh MS. Early developmental delay in children with autism: A study from a developing country. Infant Behav Dev 2015, 39: 118–123. 25827390 10.1016/j.infbeh.2015.02.017

[37] Sipes M, Matson JL. Factor structure for autism spectrum disorders with toddlers using DSM-IV and DSM-5 criteria. J Autism Dev Disord 2014, 44: 636–647. 23979069 10.1007/s10803-013-1919-3

[38] Peixoto RT, Wang W, Croney DM, Kozorovitskiy Y, Sabatini BL. Early hyperactivity and precocious maturation of corticostriatal circuits in Shank3B (-/-) mice. Nat Neurosci 2016, 19: 716–724. 26928064 10.1038/nn.4260 PMC4846490

[39] Cheng PL, Poo MM. Early events in axon/dendrite polarization. Annu Rev Neurosci 2012, 35: 181–201. 22715881 10.1146/annurev-neuro-061010-113618

[40] Gao FB, Brenman JE, Jan LY, Jan YN. Genes regulating dendritic outgrowth, branching, and routing in *Drosophila*. Genes Dev 1999, 13: 2549–2561. 10521399 10.1101/gad.13.19.2549 PMC317067

[41] Miller M. Maturation of rat visual cortex. I. A quantitative study of Golgi-impregnated pyramidal neurons. J Neurocytol 1981, 10: 859–878. 6171624 10.1007/BF01262658

[42] Casanova EL, Casanova MF. Genetics studies indicate that neural induction and early neuronal maturation are disturbed in autism. Front Cell Neurosci 2014, 8: 397. 25477785 10.3389/fncel.2014.00397 PMC4237056

[43] Maximo JO, Cadena EJ, Kana RK. The implications of brain connectivity in the neuropsychology of autism. Neuropsychol Rev 2014, 24: 16–31. 24496901 10.1007/s11065-014-9250-0 PMC4059500

[44] Persico AM, Bourgeron T. Searching for ways out of the autism maze: Genetic, epigenetic and environmental clues. Trends Neurosci 2006, 29: 349–358. 16808981 10.1016/j.tins.2006.05.010

[45] Bourgeron T. A synaptic trek to autism. Curr Opin Neurobiol 2009, 19: 231–234. 19545994 10.1016/j.conb.2009.06.003

[46] Lainhart JE, Piven J, Wzorek M, Landa R, Santangelo SL, Coon H, *et al*. Macrocephaly in children and adults with autism. J Am Acad Child Adolesc Psychiatry 1997, 36: 282–290. 9031582 10.1097/00004583-199702000-00019

[47] Fombonne E, Rogé B, Claverie J, Courty S, Frémolle J. Microcephaly and macrocephaly in autism. J Autism Dev Disord 1999, 29: 113–119. 10382131 10.1023/a:1023036509476

[48] Kelleher RJ 3rd, Bear MF. The autistic neuron: Troubled translation? Cell 2008, 135: 401–406. 18984149 10.1016/j.cell.2008.10.017

[49] Phillips M, Pozzo-Miller L. Dendritic spine dysgenesis in autism related disorders. Neurosci Lett 2015, 601: 30–40. 25578949 10.1016/j.neulet.2015.01.011 PMC4496332

[50] Li YX, Zhang Y, Lester HA, Schuman EM, Davidson N. Enhancement of neurotransmitter release induced by brain-derived neurotrophic factor in cultured hippocampal neurons. J Neurosci 1998, 18: 10231–10240. 9852560 10.1523/JNEUROSCI.18-24-10231.1998 PMC6793341

[51] Rostami J, Holmqvist S, Lindström V, Sigvardson J, Westermark GT, Ingelsson M, *et al*. Human astrocytes transfer aggregated alpha-synuclein via tunneling nanotubes. J Neurosci 2017, 37: 11835–11853. 29089438 10.1523/JNEUROSCI.0983-17.2017 PMC5719970

[52] Kharazia VN, Weinberg RJ. Tangential synaptic distribution of NMDA and AMPA receptors in rat neocortex. Neurosci Lett 1997, 238: 41–44. 9464650 10.1016/s0304-3940(97)00846-x

[53] Nusser Z, Lujan R, Laube G, Roberts JD, Molnar E, Somogyi P. Cell type and pathway dependence of synaptic AMPA receptor number and variability in the hippocampus. Neuron 1998, 21: 545–559. 9768841 10.1016/s0896-6273(00)80565-6

[54] Gomperts SN, Rao A, Craig AM, Malenka RC, Nicoll RA. Postsynaptically silent synapses in single neuron cultures. Neuron 1998, 21: 1443–1451. 9883736 10.1016/s0896-6273(00)80662-5

[55] Chen LW, Tse YC, Li C, Guan ZL, Lai CH, Yung KK, *et al*. Differential expression of NMDA and AMPA/KA receptor subunits in the inferior olive of postnatal rats. Brain Res 2006, 1067: 103–114. 16376317 10.1016/j.brainres.2005.10.054

[56] Liao D, Zhang X, O’Brien R, Ehlers MD, Huganir RL. Regulation of morphological postsynaptic silent synapses in developing hippocampal neurons. Nat Neurosci 1999, 2: 37–43. 10195178 10.1038/4540

[57] Liao D, Scannevin RH, Huganir R. Activation of silent synapses by rapid activity-dependent synaptic recruitment of AMPA receptors. J Neurosci 2001, 21: 6008–6017. 11487624 10.1523/JNEUROSCI.21-16-06008.2001 PMC6763128

[58] Kerchner GA, Nicoll RA. Silent synapses and the emergence of a postsynaptic mechanism for LTP. Nat Rev Neurosci 2008, 9: 813–825. 18854855 10.1038/nrn2501 PMC2819160

[59] Isaac JT, Nicoll RA, Malenka RC. Evidence for silent synapses: Implications for the expression of LTP. Neuron 1995, 15: 427–434. 7646894 10.1016/0896-6273(95)90046-2

[60] Liao D, Hessler NA, Malinow R. Activation of postsynaptically silent synapses during pairing-induced LTP in CA1 region of hippocampal slice. Nature 1995, 375: 400–404. 7760933 10.1038/375400a0

[61] Durand GM, Kovalchuk Y, Konnerth A. Long-term potentiation and functional synapse induction in developing hippocampus. Nature 1996, 381: 71–75. 8609991 10.1038/381071a0

[62] Petralia RS, Esteban JA, Wang YX, Partridge JG, Zhao HM, Wenthold RJ, *et al*. Selective acquisition of AMPA receptors over postnatal development suggests a molecular basis for silent synapses. Nat Neurosci 1999, 2: 31–36. 10195177 10.1038/4532

[63] Ha HTT, Leal-Ortiz S, Lalwani K, Kiyonaka S, Hamachi I, Mysore SP, *et al*. Shank and zinc mediate an AMPA receptor subunit switch in developing neurons. Front Mol Neurosci 2018, 11: 405. 30524232 10.3389/fnmol.2018.00405 PMC6256285

[64] Bariselli S, Tzanoulinou S, Glangetas C, Prévost-Solié C, Pucci L, Viguié J, *et al*. SHANK3 controls maturation of social reward circuits in the VTA. Nat Neurosci 2016, 19: 926–934. 27273769 10.1038/nn.4319 PMC4948673

[65] Niescier RF, Lin YC. The potential role of AMPA receptor trafficking in autism and other neurodevelopmental conditions. Neuroscience 2021, 479: 180–191. 34571086 10.1016/j.neuroscience.2021.09.013

[66] Purcell AE, Jeon OH, Zimmerman AW, Blue ME, Pevsner J. Postmortem brain abnormalities of the glutamate neurotransmitter system in autism. Neurology 2001, 57: 1618–1628. 11706102 10.1212/wnl.57.9.1618

[67] Akhondzadeh S, Tajdar H, Mohammadi MR, Mohammadi M, Nouroozinejad GH, Shabstari OL, *et al*. A double-blind placebo controlled trial of piracetam added to risperidone in patients with autistic disorder. Child Psychiatry Hum Dev 2008, 39: 237–245. 17929164 10.1007/s10578-007-0084-3

[68] Mei Y, Monteiro P, Zhou Y, Kim JA, Gao X, Fu Z, *et al*. Adult restoration of Shank3 expression rescues selective autistic-like phenotypes. Nature 2016, 530: 481–484. 26886798 10.1038/nature16971 PMC4898763

