# Supplementary material for: Deciphering the Role of Shank3 in Dendritic Morphology and Synaptic Function Across Postnatal Developmental Stages in the Shank3B KO Mouse
Source: Neurosci Bull. 2024 Dec 18;41(4):583–99. doi: 10.1007/s12264-024-01330-y (PMC11978597; doi:10.1007/s12264-024-01330-y)
Supplement: Supplementary file 1 — Supplementary file1 (PDF 370 KB) [file 12264_2024_1330_MOESM1_ESM.pdf]

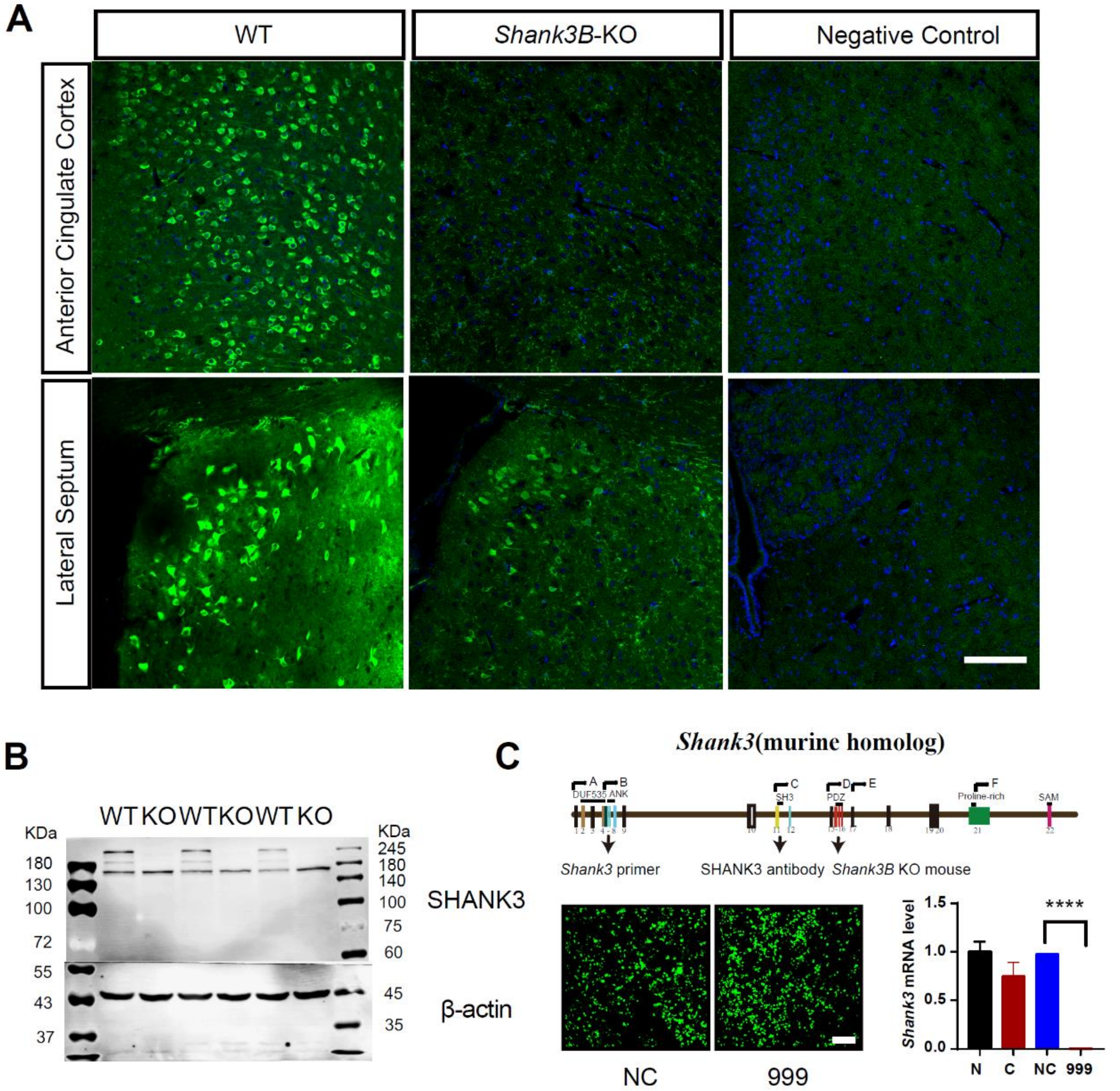

Fig. S1 Validation of SHANK3 antibody specificity.

A Validation of SHANK3 antibody specificity with immunofluorescence in the ACC (upper) and lateral septum (lower). Negative Control: only the fluorescent secondary antibody is applied without the primary antibody. Scale bar, 100 µm. **B** Validation of SHANK3 antibody specificity with Western blot. **C** Diagram of the *Shank3* mRNA structure in mice (upper); arrowheads pinpoint the target sequences for the *Shank3* primer, SHANK3 antibody, and the *Shank3B* KO mouse model. The accompanying image (left) displays the plasmid expression in Neuro2a cell lines, followed by a statistical analysis charting the relative *Shank3* mRNA expression levels across various experimental groups (right). The groups are defined as follows: N, the normal cell line without transfection reagents; C, cells with transfection reagents but no plasmid; NC, cells containing the control shRNA plasmid; and 999, cells with the Shank3 shRNA plasmid. Scale bar, 100 µm.
